# Institutional Needs

**Published:** 1913-02-01

**Authors:** 


### Institutional Needs.

### MAPPING THE ACT.

All who care for intellectual clarity will be glad to learn that the Association of Standardised Knowledge, Limited, of 15-16 Buckingham Street, Adelphi, London, W.C., have grouped by "a scientific method of functional analysis" the whole workings of the Insurance Act and presented them in chart form. The chart, among other things, is claimed as an index and commentary of the Act, and in a Special Book Number, which will be published on February 15, we hope to review it in detail.

### A STANDARD DRINK.

Cocoa probably is the most popular form of nutrient beverage which science and human ingenuity have been able yet to discover. The popularity of chocolate as a sweet paved the way for the acceptation of cocoa, which partly, because it is adapted for taking in liquid form, is a much more active stimulant. It has now become one of the standard foods of the nation, and Mr. Sandow has made an interesting attempt to standardise not merely its popularity but its purity. His "Health and Strength Cocoa" is certified as being entirely free from added alkali. This point is important, for the various processes for manufacturing, treating, or preserving foodstuffs often purchase length of life at the cost of what is simply disguised adulteration. Sandow's "Health and Strength Cocoa" has a right to the careful examination of every institution and practitioner, so that its qualities may receive the unimpeachable testimony of repeated expert experience in the kitchens of the hospital, the doctor, and the patient's home. The importance of a pure, carefully-prepared cocoa is so great that no one can afford to pass by Sandow's "Health and Strength Cocoa" without a personal trial of its merits.

